# Seroprevalence of syphilis and its risk factors among pregnant women attending antenatal care at Felege Hiwot Referral Hospital, Bahir Dar, northwest Ethiopia: a cross-sectional study

**DOI:** 10.1186/s13104-019-4106-6

**Published:** 2019-01-31

**Authors:** Kiros Tareke, Abaineh Munshea, Endalkachew Nibret

**Affiliations:** 1 Biology Department, College of Science, Bahir Dar University, P.O. Box-79, Bahir Dar, Ethiopia; 2 Biotechnology Research Institute, Bahir Dar University, P.O. Box-79, Bahir Dar, Ethiopia

**Keywords:** Pregnant women, Risk factors, Seroprevalence, Syphilis

## Abstract

**Objective:**

The objective of this study was to investigate the seroprevalence and associated risk factors of syphilis among pregnant women attending antenatal care at Felege Hiwot Referral Hospital, northwest Ethiopia.

**Result:**

Of the total 384 screened women for syphilis, 10 (2.6%) were found to be seropositive for *Treponema pallidum*. The odds of infection were about ten times (COR = 9.77, p = 0.002) higher in divorced women than in married women. The likelihood of syphilis was almost three times higher among rural residents compared to urban residents (COR = 3.48, p = 0.079). The likelihood of being infected with syphilis was about five times higher (COR = 5.25, p = 0.018) in women who had prior history of multiple sexual partners. The risk of syphilis was 4.42 (p = 0.071) and 2.67 fold (p = 0.226) greater in women with previous history of abortion and other sexually transmitted diseases (STDs), respectively. It can be concluded that a relatively low seroprevalence of syphilis was observed among the study subjects. Nevertheless, health promotion activity directed at raising the awareness of the community towards the modes of transmission of syphilis and its health impact is important in the prevention of the disease in pregnant women.

## Introduction

Syphilis is a sexually transmitted infectious disease caused by the bacterium *Treponema pallidum* and it spreads most commonly through sexual activity. In addition, it can also be transmitted from infected pregnant women to their unborn children. If syphilis is left untreated, it can lead to devastating fetal outcomes in the second or third trimesters of pregnancy [1, 2].

Despite the availability of inexpensive and effective antibiotic therapy, syphilis remains a prevalent disease in developing countries and has re-emerged as a public health threat in developed nations [3]. An estimated 36 million people are infected with syphilis worldwide, with 12 million new infections are reported every year, and of which 2 millions are pregnant women. More than half of infected women transmit the infection to their babies resulting in adverse pregnancy outcomes including early fetal death, stillbirth, preterm birth, low birth weight, neonatal death, and congenital infection in infants [4–6]. In Africa, seroprevalence of syphilis ranges from 4 to 15% among antenatal clinic attendees and it contributes to approximately 20% of prenatal deaths [7–9].

Variations in socio-demographic characteristics, sexual practices and behavior of the communities, inaccessibility to treatment of sexually transmitted diseases (STDs) and cultural practices are amongst the risk factors associated with syphilis [10–12].

Ethiopia is one of resource constrained countries in the world and shows no continuous effort in generating epidemiological data about syphilis and its associated risk factors among pregnant women. In view of this, we undertook this study to determine the seroprevalence of syphilis among pregnant women attending antenatal care at Felege Hiwot Referral Hospital (FHRH), Bahir Dar, Ethiopia.

## Main text

### Methods

#### Study design and area

Hospital based prospective cross-sectional study was conducted among pregnant women attending antenatal clinic (ANC) at FHRH. All newly registered pregnant women at ANC of the hospital were considered as a source population, while those who visited the center during sample collection period (November 2013 to June 2014) were considered as a study population.

Bahir Dar is the capital city of Amhara National Regional State and is located at 11°36′ latitude N and 37°23′ longitude E in north-western part of Ethiopia with an altitude of 1800 m. According to Amhara Bureau of Finance and Economic Development 2014 report, population of Bahir Dar city was estimated to be 284,020, of which 134,818 are males and 149,202 are females [13]. Among females, 93,174 of them are between 15 and 49 years of age (reproductive age groups).

#### Sample size estimation and sampling technique

The minimum sample size, 384 study subjects, was calculated using a single population proportion formula, with 95% confidence interval, 5% margin of error, and 50% prevalence [14]. Then, every eligible pregnant woman was included in the study through simple random sampling technique until the required sample size was obtained.

#### Data collection and processing

Questionnaire was administered to every eligible consented woman to gather data on socio-demographic characteristics, gestational age, parity status, sexual behavior, and awareness toward syphilis.

After administering the questionnaire, five milliliters of venous blood sample was drawn from each of the study participant and put into plain vacutainer tubes. Sera were then separated from the whole blood by centrifugation at 3000 rpm for 5 min and transferred into labeled tubes and kept at − 20 °C for further laboratory analysis.

Serological analyses of serum samples were carried with two types of diagnostic kits according to the manufacturers’ instructions and recommendations. The sera were initially tested with Rapid Plasma Reagin (RPR) card test to detect non-Treponemal antibodies. Positive and negative controls were included in the assays. All RPR positive sera were subjected to Immunochromatography test strips (ICS) as a confirmatory test. When RPR and ICS tests are positive, then the subject is considered as syphilis positive.

#### Data analysis and interpretation

Questionnaire and serodiagnosis data were analyzed using SPSS version 20. Chi square (χ^2^) test was conducted to determine the association between syphilis and risk factors. Then, strength of association in terms of crude odds ratio (COR) between syphilis and risk factors was analyzed using univariate logistic regression.

### Results

#### Socio-demographic variables

The mean age and age range of subjects were 26.95 and 18–44 years old, respectively. Majority (73.7%) of participants were in the age group of 20 to 29 years old. Most of them were married (93.2%) and urban dwellers (88.5%). Similar proportions of women attended high school (30.2%) and college and above (31.8%) followed by those (26.8%) who could read and write. Occupationally, 43% of the participants were housewives followed by government employees (29.4%). Most (57.8%) earned above 1000 Ethiopian Birr (ETB) per month while 19.3% earned less than 500 ETB (1 USD ≈ 20 ETB, 2014 exchange rate) (Table 1).

**Table 1:** Sociodemographic characteristics of pregnant women attending ANC at Felege Hiwot Referral Hospital, November 2013 to June 2014

Characteristics	Frequency (n)	Percentage (%)
Age categories (years)		
< 20	6	1.6
20–29	283	73.7
30–39	88	22.9
≥ 40	7	1.8
Marital status		
Single	21	5.5
Divorced	5	1.3
Married	358	93.2
Residence		
Rural	44	11.5
Urban	340	88.5
Educational status		
Illiterate	43	11.2
Read and write	103	26.8
High school	116	30.2
College and above	122	31.8
Occupational status		
Government employee	113	29.4
Private	86	22.4
House wife	165	43.0
Daily laborers	20	5.2
Monthly income (Eth Birr)		
< 500	74	19.3
500–1000	88	22.9
> 1000	222	57.8

#### Seroprevalence of syphilis across socio-demographic variables

Of the total of 384 participants, 10 (2.6%) were seropositive for syphilis. A significant association between syphilis and age categories, marital status and residence of the subjects was observed. Most of the infections were detected among 40 and above years old (28.5%) and divorced women (40%). Three fold higher (6.8%) seroprevalence was found among rural dwelling compared to urban dwelling women (2.1%) (Table 2).

**Table 2:** Socio-demographic characteristics of pregnant women in relation to their serostatus at Felegehiwot Referral Hospital, November 2013 to June 2014

Characteristics	Total examined n (%)	Serostatus for *T. pallidum*		χ^2^ (df) *p* value	COR (CI 95%)	P-value
		Positive n (%)	Negative n (%)			
Age categories (years)						
< 20	6 (1.5)	0 (0)	6 (100)		1.00	
20–29	283 (73.6)	7 (2.4)	276 (97.6)	19.53	0.02 (0.00, 0.30)	0.004
30–39	88 (22.9)	1 (1.1)	87 (98.9)	(3)	0.07 (0.01, 0.47)	0.006
≥ 40	7 (1.8)	2 (28.5)	5 (71.5)	P = 0.000	0.04 (0.00, 54)	0.015
Marital status						
Single	21 (5.5)	3 (14.3)	18 (85.7)	18.92	0.67 (0.54, 8.19)	0.751
Divorced	5 (1.3)	2 (40)	3 (60)	(2)	9.77 (2.26, 2.29)	0.002
Married	358 (93.2)	5 (1.4)	353 (98.6)	P = 0.000	1.00	
Residence						
Urban	340 (88.5)	7 (2.1)	333 (97.9)	3.47 (1)	1.00	0.079
Rural	44 (11.5)	3 (6.8)	41 (93.2)	P = 0.052	3.48 (0.86, 13.98)	
Educational status						
Illiterate	43 (11.2)	4 (9.3)	39 (90.6)	1.11 (3)	1.62 (0.26, 10.09)	0.262
Read and write	103 (26.8)	3 (2.9)	100 (97.1)	P = 0.774	2.78 (0.37, 20.38)	0.379
High school	116 (30.2)	2 (1.7)	114 (98.3)		1.93 (0.31, 11.99)	0.312
College and above	122 (31.8)	1 (0.8)	121 (99.1)		1.00	
Occupational status						
Govt. employee	113 (29.4)	1 (0.9)	112 (99.1)		1.00	
Private	86 (22.3)	3 (3.6)	83 (96.4)	5.91 (3)	0.25 (0.02, 2.41)	0.230
House wife	165 42.9)	4 (2.4)	161 (97.6)	P = 0.116	0.36 (0.04, 3.25)	0.363
Daily laborer	20 (5.2)	2 (10)	18 (90)		0.08 (0.07, 0.93)	0.044
Family monthly income (Eth Birr)						
< 500	74 (19.3)	3 (4.1)	71 (95.9)	3.29 (2)	0.32 (0.64–1.64)	0.174
500–1000	88 (22.9)	3 (3.4)	85 (96.6)	P = 0.193	0.28 (0.63, 1.13)	0.108
> 1000	222 (57.8)	4 (1.8)	218 (98.2)		1.00	

#### Medical history, sexual behavior and awareness of subjects

About 43.5%, 30.7% and 25.8% of the women had one, two and more than two pregnancies, respectively. A quarter (24.7%) of participants reported history of abortion and while 5.7% encountered stillbirths. Thirty-nine (10.2%) of the women were in their first, (35.4%) second and (54.4%) third trimesters of their pregnancies. More than 90% and 68.2% of the participants had no any previous history of STD and multi-sexual exposure, respectively. Majority (78.4%) of study subjects had awareness towards sexual transmission of syphilis while almost three-fourth (73.0%) did not know transplacental transmission of the disease. Likewise, majority (66.9%) of subjects did not clearly identify the symptoms of syphilis while the rest (33.1%) clearly knew the symptoms of the disease. About 85% of the subjects knew that STDs could be prevented through the use of condom. Majority (74.1%) of the participants’ partners did not use condom during sexual activity (Table 3).

**Table 3:** Syphilis infection in relation to clinical history, sexual behavior, and awareness of pregnant women about the disease

Characteristics	Total examined n (%)	Serostatus		X^2^ (df) P-value	COR (CI 95%)	P-value
		Positive n (%)	Negative n (%)			
Parity						
Primigravidae	167 (43.5)	3 (1.8)	164 (98.2)	5.58 (2)	1.00	
Bigravidae	118 (30.7)	5 (4.2)	113 (95.8)	P = 0.061	1.12 (0.18,6.86)	0.897
Multigravidae	99 (25.8)	2 (2.0)	97 (98.0)		0.46 (0.08,2.45)	0.368
Gestational period (stage)						
1st trimester	39 (10.2)	3 (10.3)	36 (89.7)	10.1 (2)	1.00	
2nd trimester	136 (35.4)	2 (1.5)	134 (98.5)	P = 0.006	5.58 (0.89, 34.68)	0.065
3rd trimester	209 (54.4)	5 (1.9)	205 (98.1)		3.40 (0.77,14.85)	0.104
Stillbirths						
Yes	95 (24.7)	4 (4.2)	91 (95.8)	1.28 (1)	0.48 (0.13,1.74)	0.267
No	289 (75.3)	6 (2.0)	283 (98.0)	P = 0.257	1.00	
History of abortion						
Yes	22 (5.7)	2 (9.0)	20 (91.0)	3.82 (1)	4.42 (0.88,22.21)	0.071
No	362 (94.3)	8 (2.2)	354 (97.8)	P = 0.049	1.00	
History of any STDs						
Yes	34 (8.9)	2 (5.9)	32 (94.1)	1.58 (1)	2.67 (0.54,13.11)	0.226
No	350 (91.4)	8 (2.3)	342 (97.7)	P = 0.209	1.00	
Multi-sexual exposure						
Yes	122 (31.8)	7 (5.7)	115 (94.3)	3.95 (1)	5.25 (1.33, 20.68)	0.018
No	263 (68.2)	3 (1.1)	260 (98.9)	P = 0.009	1.00	
Condom use						
Yes	99 (25.9)	2 (2.0)	97 (98.0)	0.17 (1)	1.00	0.673
No	285 (74.1)	8 (2.7)	277 (97.2)	P = 0.672	0.71 (0.14, 3.42)	
Reason not to use condom						
Partner objection	39 (13.7%)	2 (5.1%)	37 (94.9%)	1.99 (4)	2.19 (0.35, 13.65)	0.386
				P = 0.737		
Dislike using condom	70 (24.56%)	2 (2.9%)	68 (97.1%)		1.19 (0.19, 7. 33)	0.846
Ashamed to ask my partner	51 (17.89%)	2 (4%)	49 (96%)		1.65 (0.26, 10.24)	0.581
Faithful to my partner	125 (43.85%)	3 (2.4%)	122 (97.6%)		1	
Knowledge of sexual transmission						
Yes	301 (78.4)	7 (2.3)	294 (97.7)	0.42 (1)	1.00	0.517
No	83 (21.6)	3 (3.6)	80 (96.4)	P = 0.514	0.63 (0.16, 2.51)	
Knowledge of transplacental transmission						
Yes	104 (27.0)	3 (2.9)	101 (97.1)	0.04 (1)	1.00	0.738
No	280 (73.0)	7 (2.5)	273 (97.5)	P = 0.833	1.30 (0.27, 6.31)	
Knowledge about symptoms						
Yes	127 (33.1)	3 (2.4)	124 (97.6)	0.040 (1)	1.00	0.834
No	257 (66.9)	7 (2.7)	250 (97.3)	P = 0.834	1.15 (0.29,4.55)	
Knowledge of prevention of STI through condom use						
Yes	326 (84.9)	6 (1.8)	320 (98.2)	4.96 (1)	1.00	0.038
No	58 (15.1)	4 (6.9)	54 (93.1)	P = 0.833	3.95 (1.07,14.46)	

Statistically significant association was observed between syphilis and gestational period, multi-sexual exposure and previous history of still birth. The highest (10.3%) and the lowest (1.5%) prevalence of syphilis were found in pregnant women who were in their first and second trimesters. Participants who had multi-sexual exposure were more (5.7%) infected than unexposed ones (1.1%). In contrast, parity, history of STDs, habit of condom use and awareness of women towards transmission and prevention were not associated with syphilis.

#### Univariate logistic regression analysis of potential risk factors

Divorced women were 9.77 times more likely to be infected with syphilis than married ones. Similarly, the odds of syphilis were 3.48 times higher among rural residing pregnant women compared to urban dwellers. However, no associations were detected between syphilis and age, occupation, educational status, and monthly income (Table 2).

Pregnant women who were in their second and third trimesters were 5.85 and 3.40 times more likely infected with syphilis compared with those in their first trimester. Furthermore, the risk of *T. pallidum* infection was 4.42 and 2.67 times higher among those who had previous history of abortion and STIs, respectively. Pregnant women who had previous history of multi-sexual exposure were at 5.25 folds of increased risk of getting the infection compared to those women who had no exposure at all. Likewise, nearly fourfolds of risk of the disease were observed among women who had no knowledge of prevention of STDs through condom use compared to those who had the awareness. However, no significant association was observed between syphilis and the risk factors considered like parity, history of stillbirth, habit of condom use and knowledge of the study subjects toward sexual and transplacental transmission and symptoms of this disease (Table 3).

### Discussion

The seroprevalence of syphilis among pregnant women in current study was 2.6%. This is consistent with reports of two hospital based studies in Ethiopia [15, 16]. However, it is much higher than syphilis sero-positivity reported among pregnant women in Nigeria (0.3%) [11] and in Tanzania (0.5%) [17]. It is also higher than findings from India (0.36%) [18] and China (0.39%) [19]. Compared to similar studies, it is lower than prevalence of 4.3%, 5.0%, 5.8% and 7.3% reported from Botswana [20], Malawi [21], the Niger Delta, Nigeria [22] and Tanzania [23], respectively. These discrepancies in the seroprevalence of syphilis among different populations within and outside Ethiopia might be a reflection of variations in sexual practice and behavior of the communities, awareness about syphilis and difference in access to treatment of STDs, cultural practices, and also differences in the laboratory techniques employed in detecting *T. pallidum* infection.

In Several epidemiological studies found significant association between risk of syphilis and educational status, where study subjects with no education or less educational attainment were at higher risk of getting syphilis [24–27]. Contrary to this, in our study we could not find any association between educational status and *T. pallidum* seropositivity. Nevertheless, the significance of education toward avoidance of the potential risk factors cannot be ruled out.

It was found that divorced pregnant women were ten times more likely to be infected with syphilis than married women. This might be due to the tendency of divorced women to have more sexual partners. On the contrary, reports from southern [26] and central [28] Ethiopia showed no association between marital status and syphilis.

In this study, no statistically significant association was found between parity and syphilis. This is in conformity with the report of Onwuezobe et al. [29]. However, a study done at Harare Maternity Hospital reported significant increase in the risk of syphilis as the parity increased [30].

Being at the third trimester was significantly associated with the syphilis and it was shown that women at this stage of pregnancy were three times more likely to get the infection compared to those at first trimester of pregnancy. This finding could be attributed to a possible common practice where most pregnant women visit ANC later in their pregnancies.

In our study, the risk of syphilis was greater in women with previous history of abortion and other STDs. This is in line with Zhou et al. [27] and Macêdo et al. [31] who reported significant association between syphilis and prior history of induced abortion and other STDs.

The likelihood of syphilis was almost two times higher among women living in rural setting as compared to their urban dwelling counterparts. This could be due to low level of awareness of women and limited access to mass media and social networks in rural areas. Contrary to the current finding, Assefa [15] reported a relatively higher rate of this disease among urban pregnant women.

Pregnant women who had previous history of multi-sexual exposure were six times more likely to be infected with syphilis than unexposed ones. This is in agreement with the reports of Eticha et al. [28], Moges [32] and Sule [33] who noted increased odds of syphilis among women with many sexual partners as compared to those with one sexual partner.

In conclusion, the seroprevalence of syphilis in this study was relatively lower than previous studies in Ethiopia. This might be due to preventive interventions taken and improved surveillances. Prevalence of syphilis was strongly associated with variables like rural residence, divorced marital status, and history multiple sexual exposure of the participants. So, raising the awareness of the community towards safe sex practice is still important in the reduction of the prevalence of the disease not only in pregnant women but also in general population.

## Limitation

Since this is a hospital based cross-sectional study, the reported prevalence of syphilis might not reflect the actual burden of the disease at community level. The study might be susceptible to responder recall and interviewer bias regarding the risk factors.

## Abbreviations

AIDS: Acquired Immunodeficiency Syndrome
ANC: antenatal clinic
CI: confidence interval
COR: crude odds ratio
ETB: Ethiopian Birr
FHRH: Felege Hiwot Referral Hospital
HIV: human immunodeficiency virus
ICS: immunochromatography test strips
RPR: rapid plasma reagin
STIs: sexually transmitted infections
WHO: World Health Organization
